# Two-stage genome-wide association study identifies integrin beta 5 as having potential role in bull fertility

**DOI:** 10.1186/1471-2164-10-176

**Published:** 2009-04-24

**Authors:** Jean M Feugang, Abdullah Kaya, Grier P Page, Lang Chen, Tapan Mehta, Kashif Hirani, Lynne Nazareth, Einko Topper, Richard Gibbs, Erdogan Memili

**Affiliations:** 1 Department of Animal and Dairy Sciences, Mississippi State University, Mississippi State, MS 39762, USA; 2 Alta Genetics Inc. Watertown, WI, USA; 3 School of Public Health, University of Alabama-Birmingham, Birmingham, AL, USA; 4 Baylor College of Medicine, Houston, TX, USA

## Abstract

**Background:**

Fertility is one of the most critical factors controlling biological and financial performance of animal production systems and genetic improvement of lines. The objective of this study was to identify molecular defects in the sperm that are responsible for uncompensable fertility in Holstein bulls. We performed a comprehensive genome wide analysis of single nucleotide polymorphisms (SNP) for bull fertility followed by a second-stage replication in additional bulls for a restricted set of markers.

**Results:**

In the Phase I association study, we genotyped the genomic sperm DNA of 10 low-fertility and 10 high-fertility bulls using Bovine SNP Gene Chips containing approximately 10,000 random SNP markers. In these animals, 8,207 markers were found to be polymorphic, 97 of which were significantly associated with fertility (p < 0.01). In the Phase II study, we tested the four most significant SNP from the Phase I study in 101 low-fertility and 100 high-fertility bulls, with two SNPs (rs29024867 and rs41257187) significantly replicated. Rs29024867 corresponds to a nucleotide change of C → G 2,190 bp 3' of the collagen type I alpha 2 gene on chromosome 4, while the rs41257187 (C → T) is in the coding region of integrin beta 5 gene on chromosome 1. The SNP rs41257187 induces a synonymous (Proline → Proline), suggesting disequilibrium with the true causative locus (i), but we found that the incubation of bull spermatozoa with integrin beta 5 antibodies significantly decreased the ability to fertilize oocytes. Our findings suggest that the bovine sperm integrin beta 5 protein plays a role during fertilization and could serve as a positional or functional marker of bull fertility.

**Conclusion:**

We have identified molecular markers associated with bull fertility and established that at least one of the genes harboring such variation has a role in fertility. The findings are important in understanding mechanisms of uncompensatory infertility in bulls, and in other male mammals. The findings set the stage for more hypothesis-driven research aimed at discovering the role of variation in the genome that affect fertility and that can be used to identify molecular mechanisms of development.

## Background

Fertilization is a critical event at the onset of mammalian development. The widespread use of artificial insemination has revealed great variation in fertility among sires [1]. Some males display reduced fertility that can be overcome with higher semen volume for insemination, called compensable infertility, while others show an uncompensable infertility [2,3].

Uncompensable infertility defects may result from molecular defects caused by abnormalities in spermatozoal DNA, RNA, or proteins, which impair the ability of spermatozoa to interact with oocytes and induce embryonic development [4-6]. The quality of nuclear vacuoles, DNA integrity, and chromatin structure have been proposed as potential causes of uncompensable fertility defects [7-10]. However, most causes of bull subfertility are still unknown and are likely multigenic.

Recent advances in animal genome sequencing and associated technologies are providing new insights into the genomics study of gametes and embryos [11-14]. For instance, high-throughput technologies, including massively parallel expression and protein quantification, have revealed numerous differences between the spermatozoa of subinfertile and fertile males [15-17]. These phenotypes reflect, among other things, the genetic differences among the various sires. Single nucleotide polymorphisms (SNPs) which represent the most abundant genomic variation, have proved useful in studies of genes associated with human diseases (e.g., cancer, stroke, and diabetes) [18-21] and economically important traits in livestock (e.g., horse, pig, and cattle) [12,22-29]. The previous use of SNPs for fertility studies has been limited to a few markers, and their implication in male infertility has not yet been fully demonstrated [19,30-33].

The objective of the present study was to use a high-throughput and a high-density SNP array to conduct a near-genome-wide association study of bull fertility. Spermatozoa DNA were isolated from well-characterized low fertility (n = 10) and high fertility (n = 10) bulls (Phase I study) and examined for approximately 10,000 SNPs, followed by the screening of the four most significant SNPs in a larger population (101 low- and 100 high-fertility bulls; Phase II study).

## Methods

### Bull selection

Pure Holstein bulls were selected based on their fertility as previously described by Peddinti et al. [34]. Briefly, the progeny test program from Alta Genetics Inc. (Alta Advantage^® ^program) involving approximately 180 farms milking an average of 850 cows each was used to select the bulls (Alta Genetics Inc; Calgary, Alberta, Canada). This program provides certain benefits, including DNA verification of the paternity of offspring and pregnancy diagnoses by veterinary palpation, instead of relying solely on non-return rates 60–90 days after breeding. This depth of data allows an accurate determination of both male and female fertility traits. In addition, this program provides unique research materials to identify molecular markers associated with fertility.

### Definition of low- and high-fertility bulls

The fertility of bulls (Holstein), drawn from a total population of 874 bulls, is based on an average of 788 breeding, ranging from 101 to 11,997. We used the model described by Zwald et al. [35], which takes in account the breeding event, environmental factors and herd management factors that influence fertility performance of sires (i.e., effects of herd-year-month, parity, cow, days in milk, sire proven status). All these factors were adjusted using threshold models similar to the previously published models [35]. The fertility prediction of each bull was obtained using the Probit.F90 software [36] and expressed as the percent deviation (Table 1) of its conception from the average conception of all bulls. For the QTL analysis, the fertility was converted to a Z-score.

**Table 1:** Oligonucleotide probe sequences for single-nucleotide polymorphism (SNP) markers

NCBI SNP ID	Locus	Primers and detection probes
Rs29016875	C/T	Forward primer: 5'-GTCTGGTATTCCCATCTCTTTCAGA-3' Reverse primer: 5'-TTACTGATTGAAGGGCAACTGTGT-3' Probe 1: 5'-6FAM-TTTTCCACAGTTTATTGTG-3' Probe 2: 5'-VIC-TTTTCCACAGCTTATTG-3'
Rs29015574	C/T	Forward primer: 5'-ACTCTGTCTCTGAGATTCGATTCAGT-3' Reverse primer: 5'-CTGAAATCTTTCATTCCCTAGCTGATG-3' Probe 1: 5'-6FAM-CTGAAAACTCTATCTCTG-3' Probe 2: 5'-VIC-CTGAAAACTCTGTCTCTG-3'
Rs29024867	G/C	Forward primer: 5'-TGGAGGAGTTCTTTAATGCTTATAAATG-3' Reverse primer: 5'-GGAGGCACAAAATAGTTAACAGACATC-3' Probe 1: 5'-6FAM-CTAAACCGATTTGTAATC-3' Probe 2: 5'-VIC-CTAAACGGATTTGTAATC-3'
Rs41257187	C/T	Forward primer: 5'-CGAAATGGCTTCAAACCCTCTGTA-3' Reverse primer: 5'-TGTTGAAGGTGAAATCCACAGTGT-3' Probe 1: 5'-6FAM-CAGAAAGCCTATCTCC-3' Probe 2: 5'-VIC-AGAAAGCCCATCTCC-3'

### Isolation of pure sperm cells and DNA extraction

Alta Genetics Inc. (Watertown, WI) provided frozen semen straws of selected bulls. Thawed spermatozoa were then purified through a Percoll gradient, washed, counted, and pelleted for DNA isolation [34]. DNA was extracted from a pool of three different ejaculates of spermatozoa using the Puregene DNA isolation kit (Qiagen, Valencia, CA), with minor modifications. Spermatozoa were homogenized in the lysis buffer (containing 60 mM DTT and 60 μg proteinase K), incubated for 60 minutes at 55°C, and treated with RNase A (12 μg). Proteins were sedimented and DNA was subsequently precipitated using isopropanol. DNA was washed in ethanol, dissolved in TE buffer (pH 8.0), and quantified using the NanoDrop ND-1000 spectrophotometer (NanoDrop Technologies). DNA integrity was verified on an electrophoresis gel stained-agarose. DNA samples with high purity (A_260/A280 _≥ 1.8) and no degradation were used for the Phase I and Phase II studies.

### SNP genotyping (Phase I study)

The 10 K SNP Bovine Gene Chip (Affymetrix/ParAllele GeneChip; Affymetrix Inc., Santa Clara, CA) was used to genotype DNA samples (250 ng/μl) of 10 low-fertility and 10 high-fertility bulls. The experiment was carried out at Baylor College of Medicine (Houston, TX), and assays utilized molecular inversion probe (MIP) technology, allowing the multiplex detection of single base variants using a 4-color array hybridization assay [37,38]. The hybridization, washing, staining, and chip scanning procedures were performed using the standard protocol recommended by the manufacturer (Affymetrix Inc., Santa Clara, CA) of 9,919 SNPs analyzed in all DNA samples, the pass and completeness rates were 94.04% and 98.2%, respectively.

### Allelic discrimination analysis (Phase II study)

The allelic discrimination technique, based on the TaqMan technology (ABI Prism 7000 Sequence Detection System, Applied Biosystems, Foster City, CA) was performed by scientists at SeqWright, Inc. seqwright.com on DNA samples (4 ng/μl) of 101 low-fertility and 100 high-fertility bulls to validate the Phase I association study. Primer and probe sets were designed on a sequence of 501 nucleotides containing the SNP (250 nucleotides downstream and upstream of the SNP allele) using the Primer Express software (Applied Biosystems, Foster City, CA). The probes were designed and labeled with FAM (6-carboxy-fluorescein) or VIC fluorescent dyes (Applied Biosystems, Foster City, CA) to match perfectly either one of the alleles (Table 1).

PCR reactions were carried out in a total volume of 25 μl as recommended by the manufacturer. Each reaction consisted of 5 μl of sperm DNA in 20 μl Master Mix solution (Applied Biosystems, TaqMan Universal PCR Master Mix) containing primers (900 nM) and probes (200 nM). DNA samples were amplified by 40 times (2 min-50°C, 10 min-95°C, 15 sec-92°C, and 1 min 60°C). For each PCR run, negative (no-template) and positive (oligos) controls were added, and each run was preceded or followed by 1-minute incubation at 60°C to determine the background, or final levels of fluorescence, in each reaction. The base calls were made by examining all samples on the allelic discrimination graph, and the Sequence Detection Software (Applied Biosystems, Foster City, CA) was used to determine the homozygosis or heterozygosis of alleles. All samples were run in triplicates for each SNP allele.

### Bioinformatics

The major repository of SNP data in the National Center for Biotechnology Information (NCBI) database (dbSNP), combined with Entrez Genome (NCBI) as well as the Ensembl automatic analysis pipeline, were used to characterize and annotate the markers (SNPs and genes) based on the cattle genome assembly version 3.1. For each SNP, a sequence of 501 nucleotides consisting of the 250 nucleotides immediately upstream and downstream of the SNP allele, were extracted and the best hit in the Bovine 3.1 genome identified using BLASTN. We considered only the hits (i) found on the same chromosome as the query, (ii) with E-values equal or close to 0.00 and (iii) presenting at least 94% alignment with the full length of the query sequence. Furthermore, the same query sequences were used to search for putative transcription factor binding sites through the Transfac-blastX (TRANSFAC Database 7.0 for searching eukaryotic transcription factors).

### Functional analysis of integrin beta 5 (ITGB5)

#### Sperm preparation and treatment

Thawed spermatozoa were purified through a Percoll gradient as previously reported [39]. Motile spermatozoa were resuspended in the fertilization medium containing Heparin and PHE. The sperm concentration was adjusted to 50 × 10^6^/ml and incubated with or without the integrin beta 5 antibody (ITGB5; sc-5401: 5 and 20 μg/ml). As a control for structural effects of the antibody, spermatozoa (50 × 10^6^/ml) were also incubated with a nonmammalian protein (BIT) antibody (sc-33757: 20 μg/ml). After a 2-hour incubation at 38°C under 5% CO_2 _in air, spermatozoa were washed twice in fertilization medium and used to fertilize the oocytes. The motility of spermatozoa was comparable before and after the incubation period.

#### Oocyte maturation and in vitro fertilization

Bovine oocytes were purchased from Bomed Inc. (Madison, WI), and maturation took place during the transportation. These oocytes were washed and fertilized with treated and untreated spermatozoa at a final concentration of 10^6 ^spermatozoa/ml. After 18 hours of co-incubation, oocytes were collected, denuded, washed, fixed, and placed on slides for staining with 0.1% aceto-orcein on slides. The nuclear status of oocytes was observed under a microscope, and the fertilized oocytes (two extruded polar bodies and/or two pronuclei) were counted to assess the fertilization rates.

### Statistical analysis

In the Phase I study, the 20 bulls of varied fertility were typed for 9,919 SNP using Affymetrix 10 K Xba 142 2.0 array. A total of 1,712 markers fixed for a single genotype in this sample were excluded from further analysis. Markers were tested for Hardy-Weinberg Equilibrium using a 1 degree of freedom chi-square test. Markers with a p value < 0.05 were flagged as potentially out of HWE, but were used for association analysis.

The fertility of the bulls was converted to Z-scores and used as the quantitative trait for the association analysis. Analysis was conducted only at the markers' positions; no attempt at interval mapping was made. At each marker, the mean fertility, scored as a Z-score, was compared between genotypes using single marker regression. If the n for a genotype was 1, the samples were collapsed into the heterozygote. Benjamini- Hochberg FDR was calculated at each marker, adjusting 8,207 tests. A p-value less than 0.01 was set as the threshold of a significant association between the SNP marker and bull fertility; this corresponds to an FDR of 84%. Similar analyses were conducted in Phase II data.

## Results and discussion

The lack of methods to accurately predict sire fertility obliges the artificial insemination (AI) industry to keep and test hundreds of bulls. The selection of such bulls, whose fertility has been evaluated with progeny tests prior to their use in large-scale breeding programs, is costly and can take several years. Recent advances in cattle genome projects and molecular genetic technologies have increased the likelihood of identifying uncompensatory defects impairing the functions of spermatozoa. High-throughput technologies may help pinpoint relationships between a single DNA marker (i.e., SNP) and economically important traits in dairy cattle. Information about specific SNPs can enhance the efficiency of genetic selection, especially for traits that are (i) too difficult or expensive to measure in all animals and/or (ii) expressed after a long field trial (i.e., fertility).

### Determination of bulls with uncompensatory infertility

The most fertile and the least fertile pure Holstein bulls were selected from a pool of 874 available bulls with at least 300 breeding records. These were defined as the representative outliers for their corresponding groups and the scaling of fertility as the deviation from the population average fertility. The average breeding and fertility rates of the 10 low- and 10 high-fertility bulls used in the Phase I association study were 1,974 and -9.2% versus 3,540 and 6.2%, respectively. In the Phase II study, these values were 904 and -4.2% for low-fertility (100), and 994 and 3.4%, for high (101) fertility bulls (Table 2). The average differences in fertility rates between low and high fertile groups were 15.4% and 7.6% in Phase I and Phase II studies, respectively. These differences were considered significant by Amann and Hammersted, 2002 [40].

**Table 2:** Artificial insemination (A.I.) and fertility records of bulls

Bulls		A.I. Services	Fertility data
		
Fertility status	Total#	(Range)	(mean % ± sd)
	**SNP genotyping (Phase I study)**		
Low	10	785–11,450	-9.2 ± 4.6
High	10	891–9796	6.2 ± 1.9
	**Allelic discrimination (Phase II study)**		
Low	101	300–11,957	-4.2 ± 1.9
High	100	300–7,209	3.4 ± 1.1

### Identification of SNPs associated with fertility using DNA microarrays (Phase I study)

**Figure 1:**
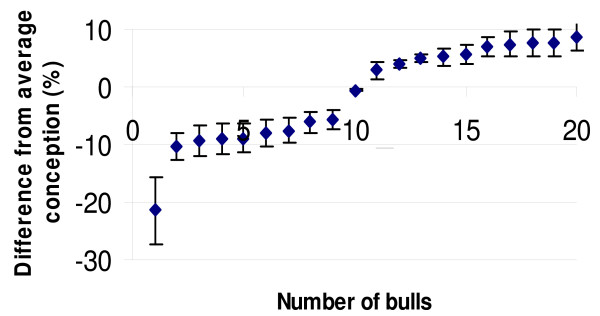
**The figure represents the fertility distribution of bulls used for SNP genotyping (mean ± SD)**. The scaling of fertility was defined as the deviation from the population average. The low-fertility bulls were scored below (negative data) the average conception rate while the high-fertility bulls scored above (positive data). The average difference between the two groups was 15.4%.

The recent progress in genomics and automation has rendered the SNP genotyping a promising technology for genetic studies of the cow genome, which contains approximately one SNP every 252 base pairs [41]. Using bovine SNP genechip microarrays (Affymetrix Inc.), we successfully genotyped 9,919 SNP markers using bull spermatozoa genomic DNA isolated from 10 low-fertility and 10 high-fertility Holstein bulls (Figure 1). Approximately 50% (4,963) of SNPs were physically located to chromosomes using the bovine genome assembly Btau-3.1 (National Center for Biotechnology Information, 2007).

**Figure 2:**
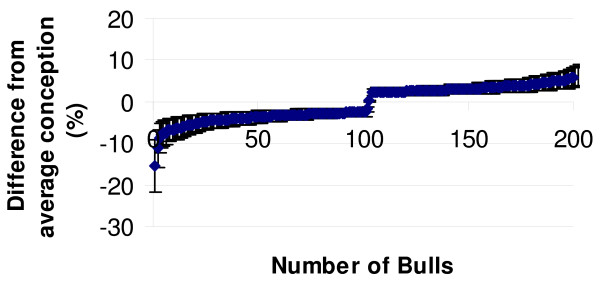
**The figure represents the distribution of p-values of the 8,207 markers analyzed in the Phase I study.** These p-values were used as guidance to select markers for Phase II.

No significant difference was found between the average of SNP call rates in both groups (95.8% ± 4.8% versus 98.6% ± 0.7%, respectively; p-value = 0.15). The HWE test revealed an FDR of 61% corresponding to 790 SNP markers that failed the test (versus the 496 expected at random), but were used for QTL analysis as mentioned above (Statistical analysis section). A total of 1,712 SNP markers were excluded from analysis because they were not polymorphic. In the Phase I samples, the fertility (Z-score) of bulls was used as the quantitative trait for the association analysis of the remaining 8,207 markers. The p-value distribution of the 8,207 markers' test showed a slight deviation from the uniform to left end (near 0) of the distribution, indicating the presence of more putative fertility-associated SNP markers in the data (Figure 2) than expected at random. Additional file 1 shows the Z-score data obtained from the four SNP markers with the highest association (p ≤ 10^-4^), and Additional file 2 shows the 97 significant markers p < 10^-2^. These most significant markers were selected to test in the Phase II samples.

High-throughput SNP arrays are powerful tools commonly used in humans to track population history and genes associated with diseases [19-21,42,43] or fertility [32,44]. The application of a such high-throughput technology has been limited in livestock [24,28,29]. The 10 K SNP Bovine Gene Chip commercialized by Affymetrix Inc. ensures a limited coverage of the cow genome (≈ 2.8 Gb, based on the Btau 3.1 genome assembly), with one SNP every 300 to 350 Kb. However, the SNP location on the array seems evenly distributed across the genome (1.18 to 2.24 SNP/cM) [45].

The extent of linkage disequilibrium (LD) around the SNPs in the Holstein population is not clear yet, but we believe the LD may extend a good distance (≤ 0.5 Mb) around the markers due to the extensive inbreeding in the population [46-48]. For example, one specific bull not in this study was found to be an ancestor 163 times, often several times in a single bull's ancestors, within 5 generations of the bulls in our study. As a result, there may be some founding effects within this study and large stretches of LD, but it is doubtful we were able to fully cover the genome as close to the maximal Fisher's Information given the marker density and coupled with the low power of this study. This suggests there are additional fertility loci to be found in the genome. The need of higher density SNP arrays (50,000 to 300,000 SNPs) has been suggested for power association and fine mapping studies in cattle [41,47,49].

### Genotyping of large numbers of bulls for the most significant SNP markers (Phase II study)

**Figure 3:**
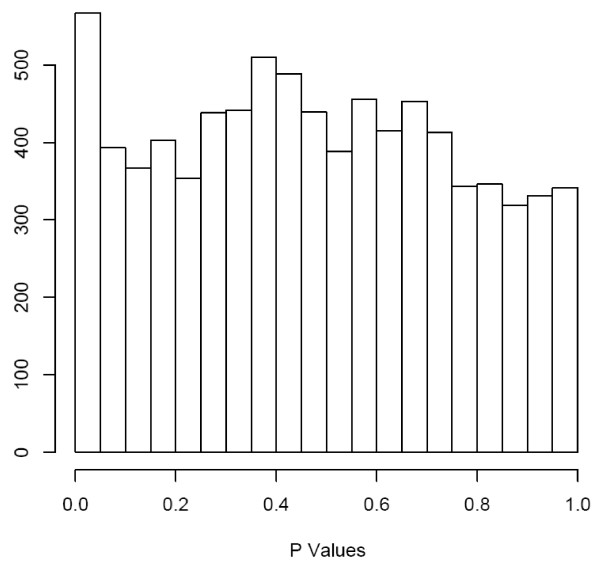
**The figure represents the fertility distribution of bulls used for allelic discrimination (mean ± SD)**. The scaling of fertility was defined as the deviation from the population average. The low-fertility bulls were scored below (negative data) the average conception rate while the high-fertility bulls scored above (positive data). The average difference between the two groups was 7.6%.

Large population sizes, often too large to be easily collected, are generally required for defining association between a given SNP and a trait with reasonable power. Thus, the use of several stages of analysis with progressively fewer markers typed in each replication population has become typical in human genome-wide association studies. The key reliability of a significant association result is replication in a second (or third) population. For example, a genome-wide-association (GWA) study of macular degeneration was successfully replicated when the initial study contained only 96 cases and 50 controls [50]. Here, we selected the four most significant SNPs from the genotyping (p ≤ 10^-4^; Phase I) study (Table 3) for replication in a larger cohort of 100 low- and 101 high-fertility bulls (Figure 3). Interestingly, the marker rs29015574 out of HWE in the Phase I study became in HWE in Phase II, as well as three other markers (HWE test > 0.05; Table 4). The fertility (quantitative trait) analysis confirmed the significant association of two of the four SNPs (rs29024867 and rs41257187; P < 0.05), while the rs29016875 tended to be significant (P = 0.09). Our calculated FDR rate for these markers (26%–63%) in the Phase I data is very close to our observed replication rate of 50%, which is known as very high for a genome wide association study [51,52].

**Table 3:** Highly informative SNP markers obtained in Phase 1 study.

SNP markers Id (rs#)	Association with fertility		Chromosome location
		
	p-values	HWE	
29016875	3.32 × 10^-5^	0.062	10
29015574	9.44 × 10^-5^	7.7 × 10^-6 ^*	9
29024867	56 × 10^-5^	0.430	4
41257187	38 × 10^-5^	0.263	1

*Indicates an out of HWE test.

**Table 4:** Overall SNP call percentages and statistics obtained in Phase II study.

Allelic variation	SNP markers			
	
	rs29016875	rs29015574	rs29024867	rs41257187
C/C	85 (42%)	128 (64%)	0 (0%)	108 (54%)
T/T	25 (12%)	6 (3%)		18 (9%)
C/T	91 (45%)	66 (33%)		74 (37%)
G/G			156 (78%)	
G/C			44 (22%)	
Total of bulls	201	200	200	200

Test for HWE	0.7566	0.6511	0.3197	0.3730
P values	0.0907	0.1853	0.0313*	0.0483*

Asterix (*) indicates significant association.

### Bioinformatics on the four highly associated SNPs

Bioinformatics were restricted on the four highest significantly associated SNPs (rs29016875, rs29015574, rs29024867, and rs41257187). Only three SNPs gave valuable information (Table 5). The SNP rs29024867 appeared as a positional marker for the collagen I alpha 2 gene, located at 2,190 base pairs of the 5' side of the SNP. The SNP rs29015574 had a single hit corresponding to a sequence similar to Rab3A-interacting molecule, which might be a potential gene candidate for fertility. The SNP rs41257187 is a synonymous polymorphism located in the exon 16 of integrin beta 5 gene (ITGB5). Each of these SNPs was located in a region (≤ 1 Mb) with low density (1–3 SNPs). Using similar SNP genechip array, Daetwyler et al., have reported a SNP frequency (SNP/cM) of 1.55, 1.75, 1.84 and 2.05 on *Bos taurus *autosome 9, 1, 4 and 10, respectively (Confer Table 3 for corresponding SNPs) [45]. This observation makes the four SNP as strong positional candidate gene markers.

**Table 5:** Bioinformatics on the four highly informative SNPs.

	SNP markers			
	
	rs29016875	rs29015574	rs29024867	rs41257187
Chromosome	10	9	4	1

Gene candidates (NCBI-BLASTN)				

Name:	Cytoplasmic dynein light chain (NW 001492841)	Rab3A-interacting molecule (NW_001495537)	Collagen I, alpha2 (NW 001494859)	Integrin β5 (NW001493888)
Length:	442/501	474/501	500/501	76/78
Identity:	96%	99%	99%	98%
E-Value:	9 × 10^-122^	0.00	0.00	4 × 10^-32^

Transcription factors (TRANSFAC-BLASTX)				

	No hits found	MEF-2D (Xl; T-01771)	Ceh-24 (S.Cb; T-03376)	No hits found
		Irx-3 (Mm; T-02439)	RAR-β3 (Mm; T-01339)	

S.Cb, Xl, and Mm stand for S. Caenorhabditis Briggsae, Xenopus laevis, and Mus musculus, respectively.

Irx, Ceh, and RAR stand for Iroquois homeobox, Caenorhabditis elegance homeodomain factor, and Retinoic acid receptor, respectively.

Putative binding sites for transcription factors with roles in embryogenesis were found within the nucleotide sequences flanking the SNPs rs29015574 (MEF-2D and Irx for, myocyte enhancer factor-2D and Iroquois homeobox, respectively) and rs29024867 (RAR β3 and Ceh-24 for, retinoic acid receptor and Caenorhabditis elegans-24, respectively). Hence, our findings suggest potential roles of the SNP mutations on regulation of gene expressions as already suggested in other studies [53-56].

Taken together, our results provide novel loci candidates whose associations with the bull fertility have not previously been reported. Because of the great interest of the non-synonymous SNP rs41257187 associated with the ITGB5, we performed a Six Frame Translation of ITGB5 mRNA reference sequence reported on NCBI (NM_174679.2) using the Baylor College of Medicine's HGSC Search Launcher to find the reading frame leading to the amino acid change. We found that the complete ITGB5 protein could be obtained from two different reading frames (+3 or +1) when using the whole length of RNA sequence or the length from the start codon (ATG), respectively. Contrary to the NCBI report, we found that the SNP mutation site (C/T) induced a synonymous amino acid change of a Proline (CCC) to another Proline (CCT) at the position 778 (P778P). The same SNP (rs41257187) inducing a synonymous amino acid change (Proline to Proline) is also reported on the Ensembl database.

The interest of integrin beta 5 in our study resided in the role(s) played by the integrin family members during fertilization and embryogenesis. Integrins are known to be expressed in a variety of tissues, including reproductive tissues of mice, humans, pigs and cattle [57-62].

### Functional analysis of integrin beta 5

The biological function of the integrin beta 5 subunit is not clearly defined. We tested the potential involvement of ITGB5 isoform on sperm-egg interaction, and the results showed that the percentages of matured oocytes (metaphase II) undergoing a successful fertilization (1–2 PN) with spermatozoa pre-exposed to anti-integrin beta 5 antibody were decreased: 74, 60, and 47% normally fertilized oocytes in the presence of 0, 5, and 20 μg ITGB5/ml, respectively. This dose-dependent inhibition was not attributed to the antibody itself since the pre-exposure of spermatozoa to the non-mammalian protein (BIT) antibody had no effect on the ability of spermatozoa to fertilize the oocytes (Table 6).

**Table 6:** Effect of masking spermatozoa ITGB5 protein during fertilization.

		Total	Unfertilized	Fertilized	Not
Groups		Oocytes (N)	Oocytes N (%)	Normally N (%)	Determined N (%)

Anti-ITGB5*	0	330	45 (13)^a^	242 (74)^a^	43 (13)^a^
(μg/ml)	5	313	106 (34)^b^	190 (60)^ab^	17 (5)^a^
	20	293	116 (40)^b^	137 (47)^b^	40 (14)^a^
Anti-BIT* (μg/ml)	20	263	45 (17)^a^	190 (72)^a^	28 (11)^a^

*Bovine spermatozoa were exposed to antibodies (anti-ITGB5 and anti-BIT) for 2 h before being using to fertilize the oocytes.

"Fertilized oocytes" refers to oocytes observed with at least one pronucleus, while "Unfertilized oocytes" refers to those without any pronuclei, but containing a Germinal Vesicle or Metaphase-1 or -2 structures (with only one extruded polar body).

(%) Percentage expressed on total oocytes.

^a, b ^Values with different superscripts within the same columns are significantly different (Student's test; p < 0.05).

We ruled out the likelihood of non-specific interactions of the anti-ITGB5 antibody and other integrin beta subunits. Indeed, the antibody is raised against a specific N-terminal epitope, which amino acid sequence is not found in other subunits of integrin beta protein. Therefore, our results contribute to the growing body of reports supporting the presence of αvβ integrins on sperm membranes [62,63]. Their putative differential expression in subfertile and fertile bulls could be used as markers for fertility, as already suggested for the αv6β3 integrin in humans [64]. Furthermore, our results contrast with the main body of literature reporting the presence of integrins mainly on the oocyte membrane while their ligands, ADAM family proteins, are on the sperm membrane [57,58,60,61,65]. In addition to this structural receptor function of integrins at fertilization (participation in cell-cell and cell-matrix interactions), integrin beta 5 might serve as a signaling receptor that induces serial events (such as inositol lipid turnover and protein phosphorylation) in the sperm, affecting fertilization and early embryo development.

## Conclusion

We have employed a high-density SNP genome association study to identify loci that may play a role in fertility in dairy cattle and significant results in two of four loci were replicated in a second population. Functional studies, including one for genes harboring a replicated locus, the ITGB5 gene, suggest that it may play a role in sperm-egg interaction. These results provide a foundation for more hypothesis-driven research in genome biology, evolution, QTL mapping, and discovering genes and genomic regions for selectable traits.

## Authors' contributions

JMF carried out the sperm DNA isolation, performed the bioinformatics and functional analyses, and participated in the manuscript writing. AK and ET carried out the collection and analysis of fertility data and contributed to the study design and manuscript writing. GPP, LC, and TM participated in the design of the study and performed the statistical analysis, the SNP genotyping, and allelic discrimination data, and participated in manuscript writing. KH, LN, and RG performed the SNP genotyping and contributed to the manuscript writing. EM participated in the design of the study, coordinated the study and participated in manuscript writing.

## Supplementary Material

Additional File 1 **Table S1**. Mean fertility (in Z-score), standard deviation, minimum and maximum values for the four markers from the Phase II study. Click here for file

Additional File 2 **Table S2**. List of 97 SNP markers associated with fertility (P < 0.01). Click here for file
