# Supplementary material for: Two-stage genome-wide association study identifies integrin beta 5 as having potential role in bull fertility
Source: BMC Genomics. 2009 Apr 24;10:176. doi: 10.1186/1471-2164-10-176 (PMC2684547; doi:10.1186/1471-2164-10-176)
Supplement: Additional File 1 — Table S1. Mean fertility (in Z-score), standard deviation, minimum and maximum values for the four markers from the Phase II study. [file 1471-2164-10-176-S1.doc]

Supplementary Table 1. Mean fertility (in Z-score), standard deviation, minimum and maximum values for the 4 markers from the phase 2 study.

| **rs29016875** | **N** | **Mean** | **Std Dev** | **Minimum** | **Maximum** |
| --- | --- | --- | --- | --- | --- |
| C/C | 86 | 0.0806628 | 1.5361376 | -2.7715000 | 2.6170000 |
| C/T | 89 | -0.4623854 | 1.8067345 | -6.2877000 | 2.3322000 |
| T/T | 25 | -0.0639080 | 1.4491541 | -2.1696000 | 2.0636000 |
|  |  |  |  |  |  |
| **rs29015574** | **N** | **Mean** | **Std Dev** | **Minimum** | **Maximum** |
| C/C | 127 | -0.1848575 | 1.7274822 | -6.2877000 | 2.6170000 |
| C/T | 65 | -0.0441246 | 1.5515958 | -3.5127000 | 2.2029000 |
| T/T | 6 | -1.3513000 | 1.4415262 | -3.0381000 | 1.1810000 |
|  |  |  |  |  |  |
| **rs29024867** | **N** | **Mean** | **Std Dev** | **Minimum** | **Maximum** |
| C/C | 1 | 1.0376000 | . | 1.0376000 | 1.0376000 |
| G/C | 45 | -0.6547711 | 1.7871592 | -6.2877000 | 2.3322000 |
| G/G | 154 | -0.0479604 | 1.6101554 | -3.5127000 | 2.6170000 |
|  |  |  |  |  |  |
| **rs41257187** | **N** | **Mean** | **Std Dev** | **Minimum** | **Maximum** |
| C/C | 107 | -0.4434972 | 1.7342943 | -6.2877000 | 2.2029000 |
| C/T | 74 | 0.1741216 | 1.5516147 | -3.1673000 | 2.6170000 |
| T/T | 18 | -0.1395333 | 1.4971554 | -1.8990000 | 2.3322000 |
